# Phase 1 pharmacokinetic study of the oral pan-AKT inhibitor MK-2206 in Japanese patients with advanced solid tumors

**DOI:** 10.1007/s00280-015-2810-z

**Published:** 2015-06-24

**Authors:** Toshihiko Doi, Kenji Tamura, Yuko Tanabe, Kan Yonemori, Takayuki Yoshino, Nozomu Fuse, Makoto Kodaira, Hideaki Bando, Kazuo Noguchi, Takashi Shimamoto, Atsushi Ohtsu

**Affiliations:** National Cancer Center Hospital East, 6-5-1, Kashiwanoha, Kashiwa, Chiba 277-8577 Japan; National Cancer Center Hospital, 5-1-1, Tsukiji, Chuo-ku, Tokyo 104-0045 Japan; Exploratory Oncology Research & Clinical Trial Center, National Cancer Center, 6-5-1, Kashiwanoha, Kashiwa, Chiba 277-8577 Japan; MSD K.K., Kitanomaru Square, 1-13-12, Kudan-kita, Chiyoda-ku, Tokyo 102-8667 Japan

**Keywords:** MK-2206, pan-AKT inhibitor, Pharmacokinetics, Phase I study, Skin toxicity

## Abstract

**Purpose:**

MK-2206 is an oral, highly selective inhibitor of AKT. The safety, tolerability, pharmacokinetics (PK), and anti-tumor activity of MK-2206 was evaluated in Japanese patients with advanced solid tumors.

**Methods:**

Patients received a single oral dose of MK-2206 according to an every other day (QOD) dosing schedule or a once weekly (QW) dosing schedule in repeating 28-day treatment cycles, with a 7-day rest after only the first cycle. The dose-limiting toxicities (DLTs) were evaluated during Cycle 1. Full PK sampling was performed during Cycle 1.

**Results:**

Twenty-four patients were treated at 45 mg (*n* = 3) or 60 mg (*n* = 9) QOD or at 135 mg (*n* = 3) or 200 mg (*n* = 9) QW. One patient experienced a DLT at 60 mg QOD, and three patients experienced DLTs at 200 mg QW. No DLTs were observed at 45 mg QOD or at 135 mg QW. The DLTs included mucosal inflammation, hyponatremia, face edema, erythema multiforme, and hyperglycemia. Common adverse events related to MK-2206 included rash, an elevated insulin c-peptide level, stomatitis, pyrexia, eosinophilia, leukopenia, and hyperglycemia. PK differences in MK-2206 exposure were observed between Japanese patients and non-Japanese patients. The higher exposure in Japanese patients was likely caused by the relatively lower weight of Japanese patients versus non-Japanese patients. No tumor responses were observed, but six patients exhibited stable disease lasting longer than 4 months.

**Conclusions:**

MK-2206 has an acceptable safety profile in Japanese patients with advanced solid tumors and warrants further investigation.

## Introduction

AKT, also known as a protein kinase B (PKB), is a serine–threonine kinase that exists in three different isoforms: AKT1, AKT2, and AKT3 [1]. AKT is a key regulator of the phosphatidylinositol 3-kinase (PI3K)/AKT/mammalian target of rapamycin (mTOR) signaling pathway and is important for promoting cell survival and inhibiting apoptosis [1]. AKT is frequently activated in many human solid tumors as a consequence of overexpression or activating mutations of receptor tyrosine kinases, PI3K and Ras, the inactivation of tumor suppressor PTEN, and the amplification or mutation of AKT itself [1]. Thus, AKT is a crucial component of the PI3K/AKT/mTOR signaling pathway and is considered an attractive target for the development of new anticancer drugs.

MK-2206 is an oral, highly selective inhibitor of AKT that binds at a site in the pleckstrin homology (PH) domain, distinct from the ATP-binding pocket, resulting in a conformational change that prevents the localization of AKT to the plasma membrane and its subsequent activation [2]. MK-2206 inhibited purified recombinant AKT isoforms, AKT1, AKT2, and AKT3, with an in vitro 50 % inhibitory concentration [IC50] of 8, 12, and 65 nM, respectively [3]. In several cancer cell lines, MK-2206 potently inhibited AKT1 kinase activity (IC50 ≈ 20 nM) and blocked the AKT2 and AKT3 activities by two to sixfold less potently [4]. In in vivo preclinical models, MK-2206 showed anti-tumor activity as a single agent and enhanced anti-tumor activity in combination with standard chemotherapeutic agents or molecular targeted drugs [5].

A first-in-human phase 1 study was conducted to determine the maximum tolerated dose (MTD) of MK-2206 in non-Japanese patients with advanced solid tumors using an every other day (QOD) dosing schedule. The MTD for the QOD dosing schedule was 60 mg/day, based on safety and biomarker data. One of 20 (5.0 %) non-Japanese patients at 60 mg QOD experienced a DLT (grade 3 skin rash). The dose-limiting toxicities (DLTs) included skin rash and stomatitis. The most common drug-related adverse events included skin rash, nausea, pruritus, hyperglycemia, and diarrhea [6]. In view of the long terminal elimination half-life values of 60–80 h, a once weekly (QW) dosing schedule has also been pursued [7, 8]. The MTD for the QW dosing schedule was established at 200 mg/day. Four of 17 (23.5 %) non-Japanese patients at 200 mg QW experienced DLTs (grade 3 skin rash in three patients; grade 3 dermatitis acneiform in one patient). Significant AKT pathway blockade was observed with both continuous QOD and intermittent QW dosing of MK-2206 in serially obtained tumor and platelet-rich plasma [8].

In the present study, the safety, tolerability, and PK of MK-2206 in Japanese patients with advanced solid tumors were investigated using the QOD and QW dosing schedules. The tumor response to MK-2206 was also evaluated as an exploratory objective.

## Materials and methods

### Patient eligibility

This study was conducted based on the Declaration of Helsinki and the Guidelines for the Clinical Evaluation Methods of Anti-Cancer Drugs in Japan (Japanese Ministry of Health, Labour, and Welfare notification, dated 1 November 2005). The study was approved by the institutional review board of each study site.

The main eligibility criteria were as follows: histologically (or cytologically) confirmed diagnosis of locally advanced or metastatic solid tumors that had failed to respond to standard therapy or for which no standard therapy exists; a patient age of 20 years or older; an Eastern Cooperative Oncology Group performance status of 0 or 1; adequate hematologic, hepatic, and renal functions; and a hemoglobin A1c (HbA1c) level of 8 % or less. The exclusion criteria included the use of chemotherapy, radiotherapy, or biological therapy within 4 weeks prior to enrollment; primary or unstable central nervous system metastasis; and symptomatic ascites or pleural effusion requiring treatment. All the patients provided informed consent, and the study was conducted in accordance with current Good Clinical Practice standards. This study was registered at ClinicalTrials.gov as NCT01071018.

### Study design and evaluation

This study was an open-label, non-randomized, multi-center phase 1 study of MK-2206 in Japanese patients with locally advanced or metastatic solid tumors. This study was designed to investigate the safety and tolerability, PK, and anti-tumor activity of MK-2206 when administered according to either a QOD or QW dosing schedule.

MK-2206 was administered as an oral formulation at a dose of 45 or 60 mg QOD or at a dose of 135 or 200 mg QW. Patients took MK-2206 orally at least 2 h before or 2 h after the intake of food or a meal. Treatment was continued until disease progression or the occurrence of an unacceptable toxicity.

Patients received oral MK-2206 in repeating 28-day treatment cycles, with a 1-week rest after only the first cycle. The 28 days of Cycle 1 were regarded as the DLT evaluation period. A minimum of three and up to nine patients were enrolled at each dose level based on the toxicity probability intervals [9]. In the DLT assessments, if 0 of the 3 patients or ≤3 of the 9 patients had a DLT at the dose level, the dose level was considered to be tolerable.

Adverse events were graded using the National Cancer Institute Common Terminology Criteria for Adverse Events, version 3.0. DLT was defined as any of the following occurring during Cycle 1 of treatment: grade 4 neutropenia lasting for ≥7 days in duration; grade 3 or 4 neutropenia with a fever >38.5 °C and/or infection requiring antibiotic or anti-fungal treatment; grade 4 thrombocytopenia; or grade 3 or 4 non-hematologic toxicity, except for inadequately treated grade 3 diarrhea, grade 3 nausea and vomiting, rash, hyperglycemia, grade 3 elevated transaminases of ≤1 week in duration, and inadequately treated hypersensitivity reactions; any drug-related adverse event leading to a dose modification of MK-2206; unresolved drug-related adverse events regardless of grade that lasted for 2 weeks or more from the date of the next scheduled treatment; persistent increases in the QTc interval (QTc >60 ms from baseline and/or >500 ms); and clinically significant bradycardia.

The anti-tumor activity was evaluated at baseline and every 6 weeks according to the Response Evaluation Criteria In Solid Tumors (RECIST), version 1.0.

### Pharmacokinetics

Blood samples for the QOD dosing schedule were collected for PK analyses just before and 2, 4, 6, 10, 24, and 48 h after MK-2206 dosing on days 1 and 27 of Cycle 1. After the final dose of MK-2206 during Cycle 1, blood samples were drawn 96, 144, and 192 h postdose during the 1-week rest period. Samples were also drawn directly before MK-2206 treatment on days 7, 15, and 21. Blood samples for the QW dosing schedule were collected for PK analyses just before and 2, 4, 6, 10, 24, 48, and 96 h after MK-2206 dosing on days 1 and 22 of Cycle 1. After the final dose of MK-2206 in Cycle 1, blood samples were drawn 168 and 240 h postdose during the 1-week rest period. Samples were also drawn directly before MK-2206 treatment on days 8 and 15. The blood samples were centrifuged, and the plasma was separated and stored at −20 °C. The plasma concentrations were analyzed using high-performance liquid chromatography with tandem mass spectroscopy (HPLC–MS/MS) at Merck Research Labs, West Point, PA. Validation data revealed an adequate accuracy, precision, and specificity of the HPLC–MS/MS assay used in this study.

## Results

### Patient characteristics

Twenty-four Japanese patients with advanced solid tumors were enrolled and were evaluated in this study. Among the 24 patients who were treated, three patients were in the 45-mg QOD cohort, nine patients were in the 60-mg QOD cohort, three patients were in the 135-mg QW cohort, and nine patients were in the 200-mg QW cohort. The baseline characteristics of the patients are summarized in Table 1. The age range was 38.0–71.0 years (median: 57.0 years), and the most frequent solid tumors were colorectal cancer (25.0 %) and breast cancer (16.7 %). The median number of prior chemotherapy regimens was 3.5 (range 1.0–8.0). The median number of treatment cycles (1 cycle: 4 weeks) was 4.0 (range 2.0–6.0) for the 45-mg QOD cohort, 2.0 (range 1.0–12.0) for the 60-mg QOD cohort, 2.0 (range 2.0–4.0) for the 135-mg QW cohort, and 2.0 (range 1.0–5.0) for the 200-mg QW cohort. The patients discontinued the study treatment because of drug-related adverse events (*n* = 1, grade 2 rash), withdrawal of consent (*n* = 2), or progressive disease (*n* = 21).

**Table 1:** Baseline characteristics of the patients (*n* = 24)

Characteristics	No.
Age (year)	
Median	57.0
Range	38.0–71.0
Sex	
Male	10
Female	14
Weight (kg)	
Median	57.2
Range	37.0–83.0
ECOG Performance status	
0 1	16 8
Primary tumor	
Colorectal cancer	6
Breast cancer	4
Leiomyosarcoma	3
Cervical cancer	2
Esophageal cancer	2
Gastrointestinal stromal tumor	2
Others^a^	5
No. of prior systemic therapy	
Median	3.5
Range	1–8

^a^Ovarian cancer, renal pelvis cancer, renal cancer, mediastinal tumor, and pancreatic cancer (*n* = 1 each)

### Safety and tolerability

The QOD dosing schedule was investigated in two cohorts receiving 45 mg or 60 mg, and the QW schedule was investigated in two cohorts receiving 135 or 200 mg. The DLTs occurring during Cycle 1 (28 days) were evaluated. No DLTs were observed in the three patients in the 45-mg QOD cohort. One of the nine patients in the 60-mg QOD cohort experienced a DLT (grade 3 mucosal inflammation). The time until the onset of the DLT after the start of treatment was 28 days. For the QW dosing schedule, no DLTs were observed in the 135-mg QW cohort. Three of the nine patients in the 200-mg QW cohort experienced DLTs: one patient had grade 4 hyponatremia (time until onset, 10 days), one patient had grade 3 facial edema and grade 3 erythema multiforme (time until onset: 10 days for both DLTs), and one patient had grade 3 hyperglycemia and grade 3 erythema multiforme (time until onset: 11 days for both DLTs). The skin toxicities were manageable with topical, oral, and/or intravenous steroid therapy. No treatment-related deaths occurred in this study. One patient in the 200-mg QW cohort discontinued treatment because of grade 2 rash.

The common drug-related adverse events reported for all the treatment cycles in all the arms are summarized in Table 2. The most common hematological adverse events related to MK-2206 included eosinophilia (13/24; 54.2 %), leukopenia (12/24; 50.0 %), lymphopenia (9/24; 37.5 %), and neutropenia (9/24; 37.5 %). The most common non-hematological adverse events related to MK-2206 included rash (20/24; 83.3 %), an elevated insulin c-peptide level (16/24; 66.7 %), stomatitis (14/24; 58.3 %), pyrexia (13/24; 54.2 %), hyperglycemia (12/24; 50.0 %), diarrhea (10/24; 41.7 %), and an elevated blood creatinine level (8/24; 33.3 %).

**Table 2:** Common adverse events related to study medications

	45 mg QOD (*n* = 3)		60 mg QOD (*n* = 9)		135 mg QW (*n* = 3)		200 mg QW (*n* = 9)		Total (*n* = 24)	
	All grades	Grades 3–5	All grades	Grades 3–5	All grades	Grades 3–5	All grades	Grades 3–5	All grades	Grades 3–5
Blood and lymphatic system disorders										
Eosinophilia	2	0	6	0	1	0	4	0	13	0
Leukopenia	2	0	2	0	0	0	8	1	12	1
Lymphopenia	2	2	5	2	0	0	2	2	9	6
Neutropenia	2	1	2	0	1	0	4	0	9	1
Gastrointestinal disorders										
Stomatitis	2	0	6	0	0	0	6	0	14	0
Diarrhea	1	1	5	0	1	0	3	0	10	1
Nausea	0	0	1	0	1	0	3	1	5	1
Vomiting	0	0	0	0	1	0	3	0	4	0
General disorders and administration site conditions										
Pyrexia	1	0	7	0	0	0	5	0	13	0
Fatigue	1	0	4	0	1	0	4	0	10	0
Localized edema	0	0	0	0	0	0	4	0	4	0
Metabolism and nutrition disorders										
Hyperglycemia	1	0	3	0	2	0	6	2	12	2
Decreased appetite	1	0	1	0	1	0	4	1	7	1
Hypernatremia	0	0	1	0	0	0	4	1	5	1
Hypoalbuminemia	0	0	2	0	0	0	2	0	4	0
Hypokalemia	0	0	1	0	1	0	2	0	4	0
Skin and subcutaneous tissue disorders										
Rash	3	0	9	1	2	0	6	0	20	1
Pruritus	1	0	4	0	0	0	5	0	10	0
Dry skin	2	0	0	0	1	0	2	0	5	0
Erythema multiforme	0	0	0	0	0	0	4	2	4	2
Palmar-plantar erythrodysaesthesia syndrome	1	0	1	0	0	0	2	0	4	0
Investigations										
Insulin c-peptide increased	2	0	7	0	2	0	5	0	16	0
Blood creatinine increased	1	0	1	0	0	0	6	0	8	0
Eosinophil count increased	0	0	0	0	2	0	5	0	7	0
HbA1C increased	0	0	2	0	1	0	4	0	7	0
ALT increased	0	0	1	0	0	0	3	1	4	1

All grades of adverse events reported in four or more patients are listed

### Pharmacokinetic evaluation

**Fig. 1:**
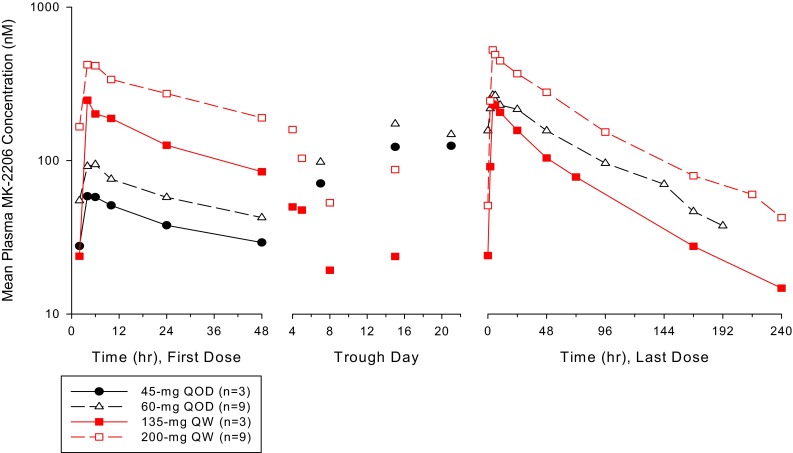
Mean MK-2206 plasma concentration profiles for Japanese patients receiving multiple oral doses of 45 or 60 mg of MK-2206 every other day (QOD) for 4 weeks or 135 or 200 mg of MK-2206 once weekly (QW) for 4 weeks (semi-log scale)

The mean serum concentration profiles for MK-2206 are shown in Fig. 1. Descriptive statistics for the PK parameters are given in Table 3. After the QOD dosing of MK-2206 (45 or 60 mg) in Japanese patients, the peak plasma concentrations were reached at a median *T*_max_ of 4–6 h. The harmonic mean terminal *t*_½_ was 79.6 and 71.8 h after QOD dosing in the 45- and 60-mg cohorts, respectively. The *C*_max_ values on Day 27 were 214 and 277 nM, respectively, while the AUC_0–48h_ values were 8490 and 9690 nM h in the 45- and 60-mg QOD cohorts, respectively. MK-2206 had accumulated at ratios of 4.59 and 3.46 on Day 27 after 45- and 60-mg QOD dosing. After the QW dosing of MK-2206 (135 and 200 mg) in Japanese patients, the peak plasma concentrations were reached at a median *T*_max_ of 4–6 h. The harmonic mean terminal *t*_½_ was 69.3 and 75.6 h after QW dosing in the 135- and 200-mg cohorts, respectively. The *C*_max_ values on Day 22 were 244 and 571 nM, respectively, while the AUC_0–48h_ values were 12,800 and 37,600 nM h in the 135- and 200-mg QW cohorts, respectively. MK-2206 had accumulated at ratios of 1.37 and 1.62 on Day 22 after QW dosing in the 135- and 200-mg cohorts, respectively.

**Table 3:** Summary of PK parameter values following multiple QOD (45 or 60 mg) or QW (135 and 200 mg) doses of MK-2206 in patients with advanced solid tumors

	AUC_t_ (nM h)^a, b^		GMR^c^	*C*_max_ (nM)^b^		GMR^c^	*C*_48-h_ (nM)^b^		GMR^c^	*T*_max_ (h)^d^		Apparent terminal *t*_½_ (h)^e^
	First dose	Last dose		First dose	Last dose		First dose	Last dose		First dose	Last dose	
45 mg QOD (*n* = 3)	1850 (41.3)	8490 (41.3)	4.59	60.5 (8.70)	214 (43.7)	3.55	28.8 (22.8)	144 (38.4)	5.02	4.0 (4.0–6.0)	6.0 (6.0–6.0)	79.6 ± 8.5
60 mg QOD (*n* = 9)	2720 (32.4)	9690 (23.7)	3.46	95.9 (33.9)	277 (26.1)	2.81	40.6 (33.5)	152 (26.1)	3.68	6.0 (2.0–10.0)	6.0 (2.0–10.0)	71.8 ± 8.9
135 mg QW (*n* = 3)	9680 (27.1)	12,800 (12.5)	1.37	234 (51.2)	244 (26.1)	1.05	83.2 (22.3)	104 (6.17)	1.25	4.0 (4.0–6.0)	6.0 (4.0–6.0)	69.3 ± 8.9
200 mg QW (*n* = 9)	24,300 (16.4)	37,600 (32.4)	1.62	439 (25.7)	571 (13.7)	1.46	186 (21.5)	302 (11.3)	1.78	4.0 (4.0–10.0)	5.0 (4.0–10.0)	75.6 ± 9.7

^a^AUC_t_ is equivalent to AUC_0–48h_ for QOD and AUC_0–168h_ for QW

^b^Geometric mean (coefficient of variation)

^c^Geometric mean ratio (GMR) determined as AUC_Day27_/AUC_Day1_ or C_Day27_/C_Day1_ for QOD, and AUC_Day22_/AUC_Day1_ or C_Day22_/C_Day1_ for QW. Ratios provided only for patients who completed dosing as planned

^d^Median (min–max)

^e^Harmonic mean ± pseudo SD

### Tumor response

As an exploratory objective, the tumor response to MK-2206 was evaluated according to the RECIST, version 1.0. Among the 24 patients who were evaluated, no tumor responses were observed. Four patients (two with cervical cancer, one with leiomyosarcoma, and one with breast cancer) had stable disease (SD) for 4 months or longer, while two patients (one with mediastinal tumor and one with esophageal cancer) had SD for 6 months or longer.

## Discussion

The primary objective of this study was to investigate the safety and tolerability of single agent MK-2206 administered as a single agent in Japanese patients with advanced solid tumors. The safety profile of MK-2206 in Japanese patients was consistent with that in non-Japanese patients. The major DLTs observed in this study were skin toxicities, which were recognized as the most common DLTs associated with MK-2206 in a previous study examining MK-2206 in non-Japanese patients [6–8]. In this study, one of nine patients experienced a DLT (mucosal inflammation) at a dose of 60 mg QOD, and three of nine patients experienced DLTs (hyponatremia, facial edema, erythema multiform, and hyperglycemia) at a dose of 200 mg QW. The skin toxicities were manageable with topical, oral, and/or intravenous steroid therapy, and most of the patients who experienced a DLT were able to resume MK-2206 treatment after recovering from the toxicities. Most of the skin rashes were maculopapular. Maculopapular rashes have been commonly reported in association with PI3K inhibitors such as BKM120, which were generally manageable with antihistamines and topical steroid therapy, while papulopustular rashes are more frequently reported in association with the EGFR inhibitors cetuximab and erlotinib and the MEK inhibitors selumetinib and trametinib [10, 11]. The other DLTs, such as hyponatremia, hyperglycemia, and facial edema, were manageable with appropriate treatment. The common adverse events related to MK-2206 included rash, an elevated insulin c-peptide level, stomatitis, pyrexia, eosinophilia, leukopenia, and hyperglycemia. These adverse events have been previously reported in association with MK-2206 in non-Japanese patients with advanced solid tumors. Based on the above findings, we concluded that the dosing schedules for MK-2206 evaluated in this study had an acceptable safety profile in Japanese patients with advanced solid tumors.

**Fig. 2:**
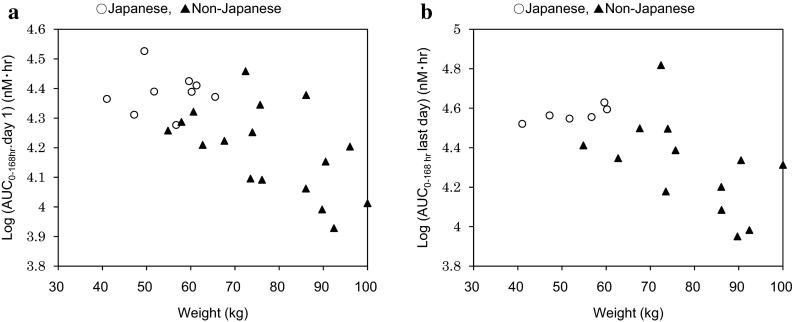
Weight versus AUC_0–168-h_ (**a** first dose: Day 1, **b** last dose: Day 22) of MK-2206 for Japanese and non-Japanese patients receiving 200 mg of MK-2206 QW

PK differences were observed after exposure to MK-2206 in Japanese patients, compared with non-Japanese patients [8]. Consequently, the PK data were analyzed using *t* tests and nonparametric Wilcoxon–Mann–Whitney tests because of potential differences in variance. The AUC, *C*_max_, and *C*_trough_ of the patients in the 200-mg QW cohort were significantly higher (*P* < 0.05) than the corresponding values in non-Japanese patients. While the exposures in individual Japanese patients overlapped with the exposures observed in non-Japanese patients receiving MK-2206, the mean exposure values (*C*_max_ and AUC) were higher (1.4–1.9-fold) in the 45- and 60-mg QOD and the 200-mg QW cohorts and lower (0.7–1.3) in the 135-mg QW cohort. The exposure to MK-2206 was found to vary according to body weight, and analyses of covariance (ANCOVA) models that adjusted for weight suggested that the PK differences between Japanese and non-Japanese patients can largely be attributed to inter-study weight differences, as the weights of the Japanese patients were significantly lower than those of the non-Japanese patients receiving 200 mg of MK-2206 (Fig. 2). The apparent terminal half-life (*t*_½_) values were similar to those observed in non-Japanese patients (compared to ~60–90 h in non-Japanese patients), suggesting no marked differences in the elimination rates between Japanese and non-Japanese patients. No differences in *T*_max_ were observed.

In this study, no tumor responses were observed, but 6 of the 24 patients (25 %) with various types of tumors experienced SD lasting longer than 4 months. MK-2206 appeared to exhibit preliminary signs of anti-tumor activity. Oncogenic PI3K pathway activation was not investigated in the patients in this study. To further characterize the anti-tumor activity of single agent MK-2206, a biomarker analysis examining the PI3K pathway in a clinical study with a large sample size will be necessary. PI3K pathway activation may be associated with resistance to chemotherapy and targeted therapy [12–15]. Considering the limited anti-tumor activity of MK-2206 monotherapy, a combination therapy containing MK-2206 may be more efficacious and should be pursued. Recently, early clinical evidence from clinical studies of MK-2206 used in combination with targeted therapy or chemotherapy has been reported [16, 17], and a clinical study including the combination of MK-2206 with a MEK inhibitor is presently ongoing in patients with advanced non-small-cell lung cancer (NSCLC) [18]. Thus, further investigation of the use of MK-2206 in combination therapies is also warranted in Japanese patients.

In conclusion, MK-2206 has an acceptable safety profile in Japanese patients with advanced solid tumors and warrants further investigation. PK differences were observed in the exposure of Japanese patients to MK-2206, compared with non-Japanese patients. These differences in exposure are likely due to inter-study differences in the body weights of the patients.
